# Tuning the metal–support interactions in transition metal-anchored heteroatom-doped graphene single-atom catalysts

**DOI:** 10.1039/d6cp00932h

**Published:** 2026-06-02

**Authors:** Angelina N. van Dam, Pascal Vermeeren

**Affiliations:** a Department of Chemistry and Pharmaceutical Sciences, AIMMS, Vrije Universiteit Amsterdam De Boelelaan 1108 1081 HZ Amsterdam The Netherlands p.vermeeren@vu.nl https://www.theochem.nl

## Abstract

Transition metal-anchored, heteroatom-doped graphene single-atom catalysts have emerged as a promising class of catalysts that combine the strengths of traditional homogeneous and heterogeneous catalysis. Strong metal–support interactions are critical for ensuring long-term catalyst stability and durability. This review assesses strategies for tuning the metal–support interactions in graphene-based single-atom catalysts, with the focus on insights from quantum chemical calculations. By systematically comparing computational studies, we address how variations in key structural and electronic features influence the metal–support bond strength. We evaluate the influence of graphene defect topology by comparing single and double vacancy sites, examine the role of heteroatom dopants (*e.g.*, B, N, or O) in the first coordination sphere, and assess periodic trends by comparing catalytically active transition metals from periods 3, 4, and 5. Overall, this review identifies key structural–electronic features governing metal–support interactions and outlines tuning handles for optimizing metal–support bond strengths.

## Abstract 2

This review demonstrates how the metal–support interactions of single-atom catalysts can be engineered by tuning vacancy size, tailoring heteroatom dopants, and selecting the catalytically active transition metal center.

## Graphical abstract

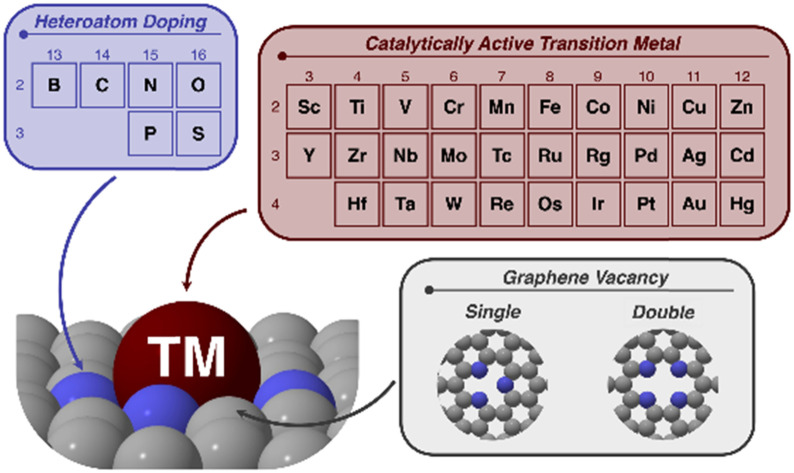

## 1. Introduction

**Fig. 1:**
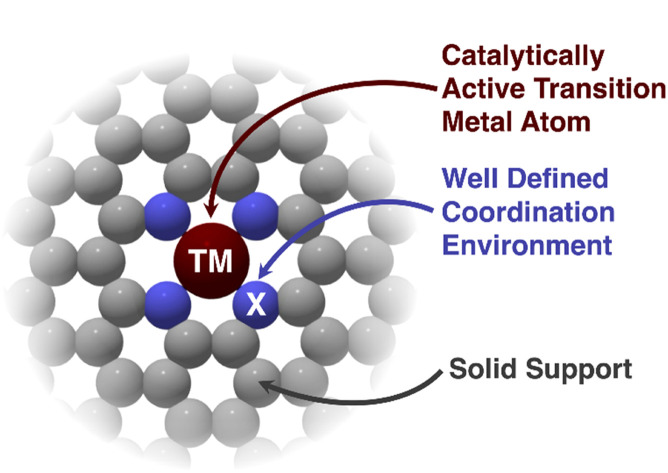
Schematic representation of the features defining graphene-based single-atom catalyst, where TM = transition metal atom and X = heteroatom doping.

**Fig. 2:**
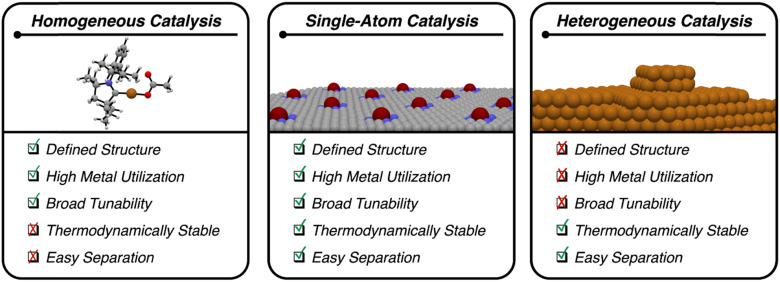
Comparison of homogeneous and heterogeneous catalysis, illustrating the unique position of single-atom catalysis.

Single-atom catalysis (SAC) is a rapidly advancing frontier in catalysis research, where individual transition metal atoms are dispersed and stabilized on solid supports to function as isolated active centers (Fig. 1).^1^ This approach maximizes metal atom utilization and provides well-defined coordination environments, enabling precise control over catalytic behavior at the atomic scale.^1*a*,*c*,2^ The principle of SAC combines the molecular specificity and electronic tunability characteristic of homogeneous catalysis with the structural stability and recoverability inherent to heterogeneous systems (Fig. 2).^1*d*,2^ Consequently, SACs bridge these two pillars in catalysis, paving the way for the next-generation of catalysts with enhanced activity, selectivity, and durability.^1*a*,*b*,*d*,2^

Graphene has emerged as a highly suitable support for SACs owing to its exceptional thermal and chemical stability, intrinsically high electronic conductivity, and large surface area that facilitates efficient dispersion of isolated catalytically active sites.^3^ In pristine form, however, the basal plane of graphene is relatively inert,^4^ and anchoring metal atoms is primarily achieved through structural defects in graphene, among which single vacancies (SV) and double vacancies (DV) play a central role.^3*i*,5^ Single vacancies, created by the removal of a single carbon atom, produce dangling bonds that strongly anchor individual metal atoms, whereas double vacancies, formed by removing two adjacent carbon atoms, generate larger coordination sites capable of stabilizing metal atoms through up to four covalent bonds. Beyond vacancy sites, heteroatom doping (*e.g.*, with N, B, or S) allows precise tuning of the metal center's first coordination sphere, thereby tailoring the stability and activity of transition metal-anchored heteroatom-doped graphene SACs.^6^ This broad tunability allows for graphene-based SACs to be employed in a wide variety of electrochemical and thermal reactions, including hydrogen evolution,^7^ nitrogen reduction,^8^ oxygen evolution,^9^ cross-coupling,^10^ and cycloaddition reactions.^11^

**Fig. 3:**
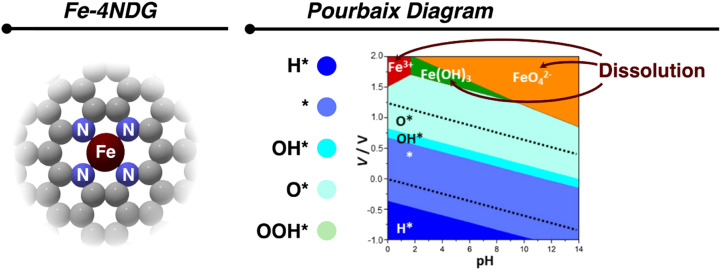
Pourbaix diagram of Fe-4NDG (indicated as *) adapted from Pacchioni *et al.*,^13^ published under the Creative Commons Attribution 4.0 License (CC BY 4.0). The pH–voltage region in which the catalyst remains stable in SAC state, either clean or covered by intermediates, is shown using colors that denote the H*, *, OH*, O*, and OOH* states. The redox levels of the H^+^/H_2_ (bottom) and O_2_/H_2_O (top) couples are marked by black dashed lines.

Catalyst stability in SACs is often hindered by demetallation of the catalytically active metal center under reaction conditions, leading to a substantial loss of active sites and hence catalytic activity.^12^ Pacchioni *et al.* employed density functional theory (DFT) to construct a thermodynamic cycle combined with Pourbaix diagrams to examine the stability of single-atom sites under electrochemical operating conditions (Fig. 3),^13^ thereby explicitly including the dynamic effects of pH and applied potential. For one of their investigated SACs, iron anchored to nitrogen-doped graphene (Fe-4NDG), the constructed Pourbaix diagram is shown in Fig. 3. In the absence of adsorbed species, denoted as *, the Fe site is predicted to be stable under hydrogen evolution reaction (HER) conditions (*V* ∼ 0 V at pH = 0), whereas at more reducing potentials the site becomes covered by hydrogen adsorbates (H*). At the equilibrium potential for the O_2_/H_2_O^−^ redox (*V* ∼ 1.23 V at pH = 0), oxygen adsorption (O*) is favored under acidic conditions, while at basic pH values the Fe atoms tend to dissolve. At oxygen evolution reaction (OER) potentials (*V* ∼ 1.5 V), dissolution dominates across almost the entire pH range from 0 to 14. By extending this analysis to a broader set of single-atom catalysts, the authors show that the electrochemical environment can either stabilize or destabilize a given catalytically active site through adsorbate formation or demetallation. Nevertheless, the decisive factor governing the resistance to demetallation is the intrinsic strength of the static metal–support interaction. Consequently, they emphasize that predictions of SAC catalytic activity should always be accompanied by a parallel assessment of catalyst stability in terms of the metal–support bond strength.

Moreover, Liu *et al.* investigated the dynamic stability of Cu–4NDG SACs under electrochemical operating conditions and demonstrated,^14^ using a constant-potential hybrid-solvation dynamics model, that hydrogen adsorption weakens the Cu–N bonds, thereby promoting Cu demetallation. These results highlight that strong metal–support interactions are essential for preventing demetallation. Both Hensen *et al.* and Senftle *et al.*,^15^ further reconciled this principle by using DFT in combination with machine learning to establish that the metal–support binding energy is a central descriptor for the activation barrier of metal atom diffusion on solid surfaces. In summary, metal–support interaction strength can be regarded as an important descriptor governing the resistance to demetallation in graphene-based SACs and hence to ensure long-term stability and durability, sufficiently strong metal–support interactions are essential.^3*i*,16^

In this review, we present an overview of strategies to tune the metal–support interactions in graphene-based SACs. Specifically, we critically assess state-of-the-art quantum chemical studies addressing the bonding between the catalytically active metal and the support, and analyze how variations in key structural and electronic features influence metal–support bond strength. Taken together, this enables the extraction of metal–support tuning handles.

**Fig. 4:**
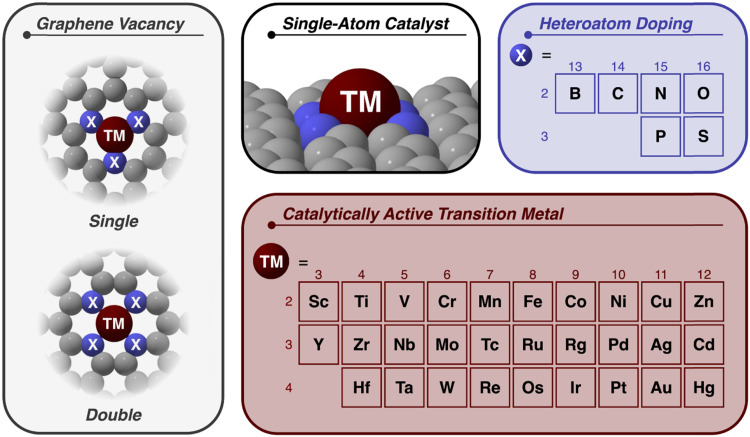
Schematic overview of three approaches to alter the stability of transition metal-anchored heteroatom-doped graphene single-atom catalysts discussed in this review.

Three primary approaches to modulate the metal–support interactions in transition metal-anchored heteroatom-doped graphene SACs are discussed (Fig. 4). First, we examine the influence of vacancy topology, with emphasis on single and double vacancy sites. Second, we explore the role of heteroatom doping, focusing on modifications within the first coordination sphere of the metal center. Finally, we assess the effect of metal identity by comparing different transition metals along periods 4, 5, and 6. Overall, we highlight the central role of local structural and electronic features in governing the metal–support interactions, while also indicating that reported trends are sometimes fragmented and, in some cases, contradictory. This suggests that the underlying stabilizing metal–support interactions are still not fully understood. Therefore, with this review, we also aim to stimulate the pursuit of a more comprehensive understanding of metal–support interactions in SACs and to support the development of an expanded and more predictive design toolbox.

The nomenclature adopted in this review is as follows: SACs are denoted as TM-3/4XDG, where TM is the catalytically active transition metal; 3 and 4 denote single- and double vacancy sites, respectively; and X indicates the dopant atom (*e.g.*, C for undoped systems, or B, N, O, P, and S for heteroatom-doped supports).

## 2. Size of the vacancy site

**Fig. 5:**
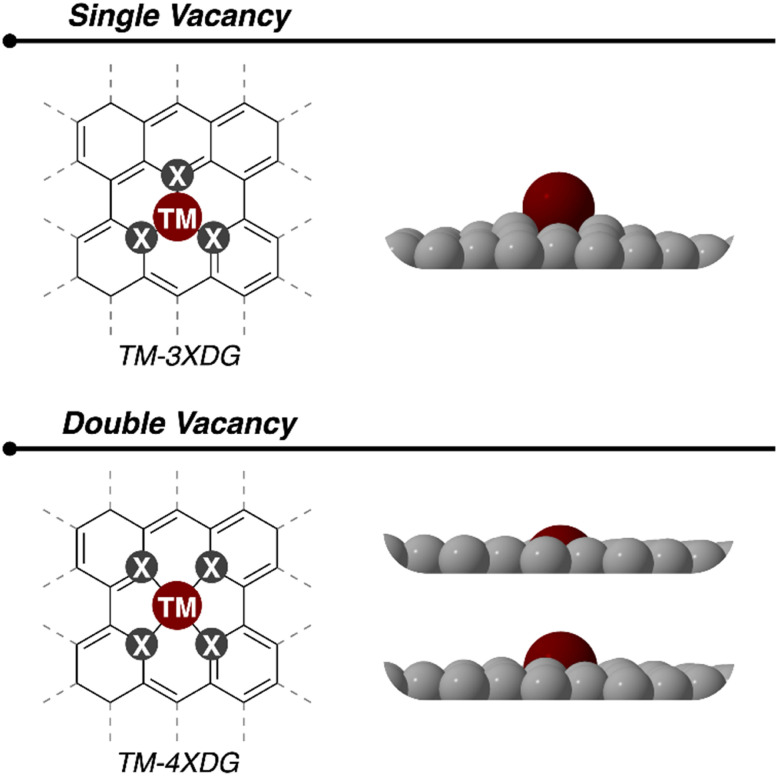
Schematic representation of transition metal atoms anchored at single vacancy (SV; TM-3XDG) and double vacancy (DV; TM-4XDG) sites in graphene. For SV sites, the TM atom is positioned above the plane of the graphene support. For DV sites, the TM atom may either lie in or protrude above the plane of the graphene support, depending on the intrinsic nature of the transition metal and its coordination environment.

In this section, we assess how the vacancy size in the graphene support influences the bonding interaction between the catalytically active transition metal and support. Here, we focus on the priory introduced vacancy sizes: single vacancy (SV; TM-3XDG) and double vacancy (DV; TM-4XDG). SV sites are produced by the removal of a single carbon atom from the graphene framework, whereas DV sites originate from the removal of two adjacent carbon atoms. For SV sites, the transition metal is typically elevated above the graphene support, whereas for DV sites, most transition metals reside within the graphene plane (Fig. 5). This was demonstrated by Di Valentin *et al.*,^17^ who analyzed the geometrical properties of Fe, Co, Ni, and Cu bound to the SV and DV sites in graphene, that is, TM-3CDG and TM-4CDG, respectively. For the TM-3CDG catalysts, all metals were positioned above the graphene plane, leading to a trigonal pyramidal coordination environment; this out-of-plane configuration was favored over in-plane placement because the SV cavity is too small to fully accommodate transition metal atoms. Accordingly, they concluded that SV sites are too small to reliably trap transition metals. The larger DV sites, on the other hand, could bind the metal in-plane of the graphene support, giving rise to a square planar coordination environment, which is structurally more compatible for a stable coordination with the transition metal.

**Fig. 6:**
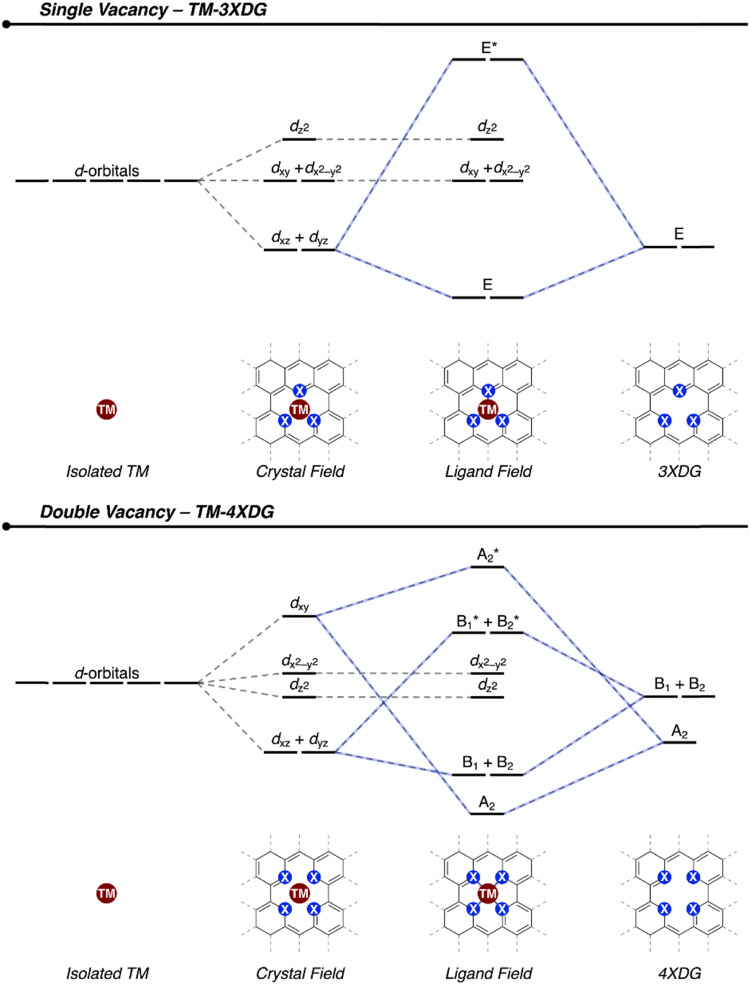
Schematic diagram of the d-orbital splitting of a transition metal in a trigonal pyramidal (single vacancy; TM-3XDG) and square planar (double vacancy; TM-4XDG) coordination environment. From left to right: degenerate d atomic orbitals of an isolated transition metal, crystal field splitting of the d atomic orbitals due to electrostatic interaction with the 3XDG/4XDG ligand, bonding, nonbonding and antibonding orbitals due to mixing of the d-orbitals with the orbitals belonging to the E irreducible representation, and B1, B2, A2 irreducible representations of 3XDG and 4XDG, respectively.

To understand the metal–support bonding interactions in TM-3XDG and TM-4XDG, a bonding scheme was proposed based in crystal and ligand field theory (Fig. 6). Previous studies by Odkhuu *et al.* demonstrated that a trigonal pyramidal coordination environment (*C*_3v_), as found for TM-3XDG, gives rise to a different ordering of the crystal field d-orbital splitting than the square planar coordination of TM-4XDG.^18^ In addition, ligand field theory predicts that the primary bonding interaction in TM-3XDG SACs arises from interactions between the metal's d_*xz*_ and d_*yz*_ atomic orbitals and the support orbitals belonging to the E irreducible representation.^19^ In contrast, for TM-4XDG SACs (*C*_2v_), in addition to bonding between the metal's d_*xz*_ and d_*yz*_ atomic orbitals and the support orbitals belonging to the B_1_ and B_2_ irreducible representations, respectively, an additional interaction is predicted between the metal's d_*xy*_ atomic orbital and support orbitals of the A_2_ irreducible representation. Together, these results qualitatively demonstrate that the nature, and hence bonding mechanism, of the metal–support interaction differs fundamentally between TM-3XDG and TM-4XDG SACs. The conclusion that DV sites are structurally preferred over SV sites is further corroborated by analogous studies on other transition metals anchoring to the SV and DV sites of SACs. Koskinen *et al.* reported similar findings for the coordination of Au to SV and DV sites in graphene.^20^ They observed that DV sites are better suited for binding Au, due to its planar structural geometry, compared to the SV sites where Au is elevated above the graphene plane. This planar geometry is preferred because it enables better overlap between the atomic orbitals of Au and the dangling bonds of the carbon atoms in the support. Consistently, Gao *et al.* also found that DV sites exhibit stronger metal–support binding interactions than SV sites, by examining TM-3CDG and TM-4CDG systems containing period 4 transition metals (TM = Ti–Cu) and the group 10 and 11 transition metals Pt, Ag, and Au.^21^ This study underscored that DV sites, owing to their larger cavity size and hence favorable coordination geometry, enable in-plane positioning and stronger metal–support bonding interactions than SV sites.

Similarly, Soldano *et al.* found that metal bonding to DV sites is stronger than to SV sites for TM-4XDG and TM-3XDG SACs, respectively. This was observed for the binding of period 4 transition metals (TM = Sc–Zn) to both the pristine undoped graphene support (X = C) and the nitrogen-doped graphene support (X = N).^22^ The origin of the stronger metal–support interactions observed for DV sites was primarily attributed to structural factors. DV sites introduce larger cavities in the support, combined with the ability to form 4 TM–X bonds instead of 3, which allows the metals–support architecture to adopt planar or near-planar geometries, thereby facilitating more effective orbital overlap between the metal and the support. In SV sites, however, metals are constrained to out-of-plane adsorption geometries, that is, being above the plane of the support, due to smaller cavity sizes. This trigonal pyramidal coordination mode reduces the metal–support bond strength, as a result of the less optimal orbital overlap.

Finally, Kim *et al.* extended these observations to a broader range of transition metals.^23^ They compared SV and DV sites for their ability to bind period 4 (TM = Sc–Zn), period 5 (TM = Y–Cd), and period 6 (TM = La–Hg) transition metals under varying degrees of nitrogen doping of the support. Regardless of the level of nitrogen doping, SV sites exhibited less favorable bonding interactions than DV sites. They observed, in line with the above-mentioned studies, that planar metal–support geometries are generally the most stable, with all SV sites resulting in transition metals positioned above the graphene support, whereas DV sites often accommodated transition metals within the plane of the support.

In contrast to the studies discussed above, Dai *et al.* reported an opposite trend, showing that Fe binds more strongly to SV sites than to DV sites.^24^ Bader charge analysis revealed significant electron transfer, establishing strong covalent bonding character between Fe and the SV site; however, in the planar Fe-4NDG configuration, an even larger amount of charge is transferred. According to the authors, the unexpectedly weaker Fe–DV interaction arises from the intrinsically higher stability of the bare DV site compared to the bare SV site. The optimized DV structure reconstructs into two five-membered rings and one eight-membered rings, which saturates the dangling bonds and thereby stabilizes this vacancy site, diminishing the propensity for Fe binding.

Zheng *et al.* likewise reported that DV anchoring sites ultimately yield less stabilizing metal–support interactions than SV sites for TM-3CDG compared to TM-4CDG SACs (TM = Sc–Zn, Pd, Pt).^25^ Although their calculations indicate a stronger initial interaction between the transition metal and the DV site, the DV site undergoes a substantial structural deformation upon metal coordination, which reduces the net binding energy and renders the overall metal–support interaction weaker for the DV compared to the SV site.

**Fig. 7:**
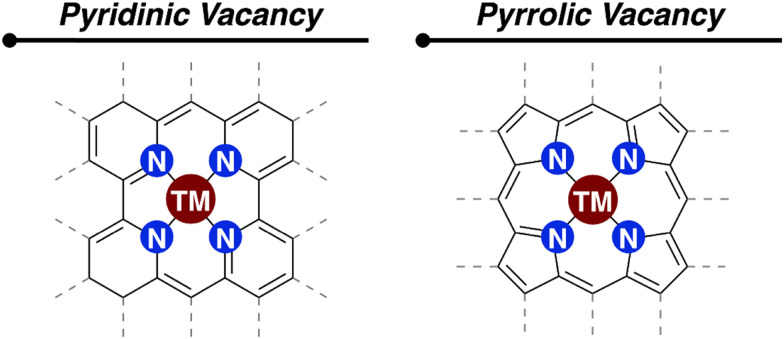
Schematic representation of transition metal atoms anchored at pyridinic vacancy (Py) and pyrrolic vacancy (Pyrr) sites in graphene.

It is worthwhile to include a brief remark on pyrrolic vacancies (Pyrr, Fig. 7), in which the composition of the vacancy site is altered. A double vacancy provides structural environments that can host either pyridinic (Py) or pyrrolic (Pyrr) nitrogen, depending on reconstruction and nitrogen incorporation site. SACs with pyrrolic vacancies have been synthesized and found to be stable.^26^ Alsunni *et al.* investigated the thermodynamic stability of SACs where period 4 (TM = Sc–Zn), 5 (TM = Y–Cd), and 6 (TM = Hf–Hg) transition metals were anchored to Py and Pyrr vacancy sites, containing varying degrees of N-doping.^27^ Based on formation energies, they found that Py sites are consistently more thermodynamically stable than Pyrr sites. This trend was further supported by the work of Fellinger *et al.*,^28^ who reported that Py sites containing Fe or Zn are more stable than their Pyrr counterparts. This difference was attributed to how well the geometry of each vacancy site matches the graphene lattice. Where Py sites integrate seamlessly into the lattice, the Pyrr vacancy introduces a geometric mismatch: the induced misalignment of the five-membered pyrrolic ring only fits the lattice when it is compensated by adjacent seven-membered-rings, thereby straining the support.

In short, comparative studies consistently show that vacancy size is a decisive factor in the stability of graphene-based SACs. SV sites are too small to anchor the transition metals within the plane of the support, resulting in out-of-plane geometries that go with a weaker metal–support binding interaction. In contrast, DV sites, with their larger and more accommodating cavities, enable a more favorable planar coordination upon binding the metal to the support. Understanding of the physical origin behind this observation remains qualitative and hence should be subjected to further investigation using quantitative theoretical models. The opposite preference for SV sites compared to DV sites reported by Dai *et al.* and Zheng *et al.* appears to stem from a different perspective, in which the pristine vacancies in the support need to deform to bind a metal, rather than directly evaluating the intrinsic metal–support bonding interaction. Summarizing, the strongest intrinsic metal–support interactions are found for metal centers embedded in the pyridinic DV site of the support, which, however, can be tuned down when going to supports with pyrrolic DV or SV sites.

## 3. Effect of heteroatom doping

**Fig. 8:**
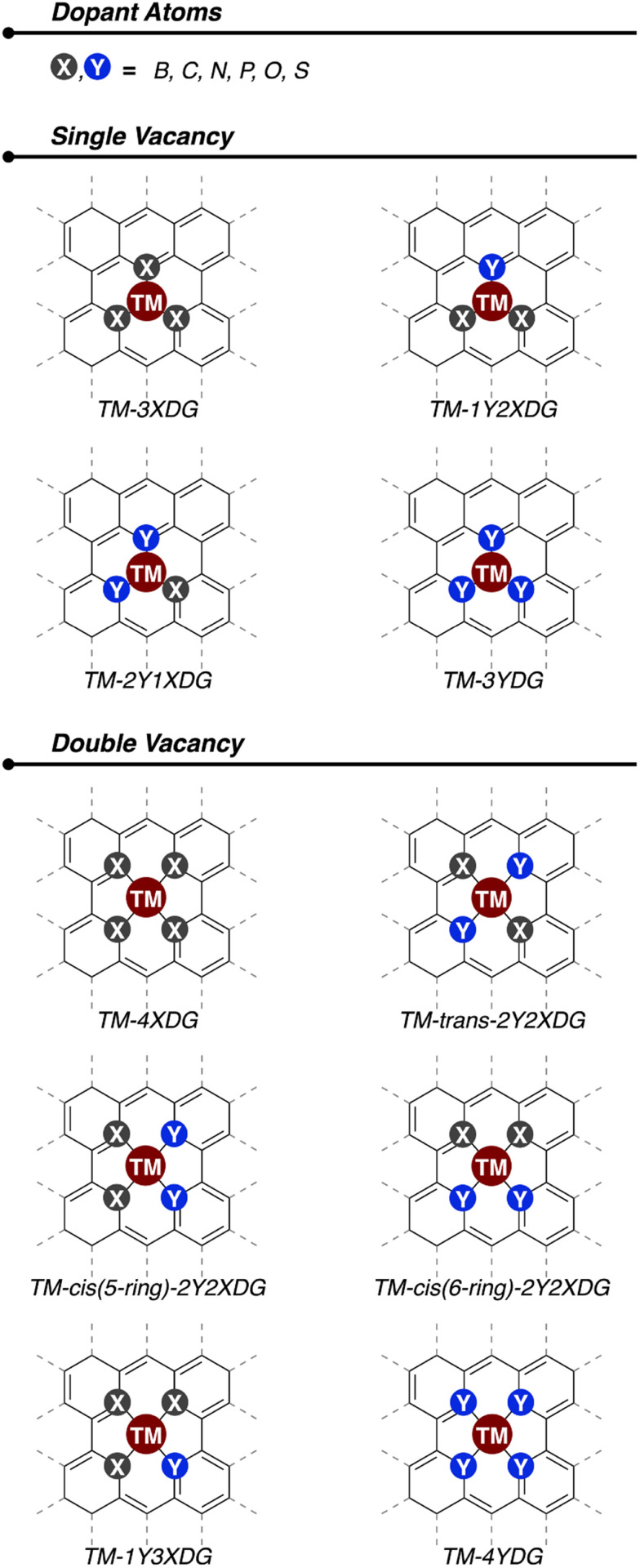
Coordination environments of transition metal single-atom catalysts in heteroatom-doped graphene supports. Illustrated are single vacancy and double vacancy configurations with varying dopant compositions.

In the next section, we evaluate the effect of heteroatom doping on the metal–support interaction in transition metal-anchored heteroatom-doped graphene single-atom catalysts (Fig. 8), starting with its effect on SV sites. For Fe-3XDG systems with varying degrees of N- and B-doping, Dai *et al.* investigated how doping influences the metal–support bond strength.^29^ Four types of doping were considered: undoped (*i.e.*, X = C), B-doped, N-doped, and N/B co-doped. The undoped system exhibited strong Fe–support bonding, whereas any form of doping weakened this interaction; moreover, increasing N- or B-doping further reduced the metal–support bonding strength. Among the dopants, N most effectively preserved the Fe center's stability relative to B- or N/B-co-doping. Density of states analysis indicated that, in undoped graphene, the Fe 3d atomic orbitals were strongly interacting with the sp^2^-hybridized orbitals on the C of the support, with the resulting Fe_3d_–C_sp^2^_ states appearing near the Fermi level, consistent with strong Fe–C interaction. In the singly B-doped SV, Fe_3d_–C_sp^2^_ states near the Fermi level were diminished due to the increased charge transfer to the unoccupied B 2p atomic orbitals, thereby reducing the Fe–support charge transfer and stability. In the singly N-doped case, Fe peaks near the Fermi level became more prominent relative to the B-doped counterpart, and Fe_3d_–C_sp^2^_ and Fe_3d_–N_sp^2^_ states were evident, indicating greater stability for N-doping. For the 2B/1N-co-doped SV, Fe 3d states decreased and shifted away from the Fermi level, demonstrating weaker Fe_3d_–X_sp^2^_ bonding states (X = B, N) and hence reflecting comparatively low stability of the SAC. Thus, the density of states analyses revealed a progressive weakening of Fe–support binding interaction from the undoped to N-doped graphene SAC and a further weakening to the B- and N/B-co-doped graphene SAC. Gaudry *et al.* reported that higher levels of N-doping in Co-3XDG, Ni-3XDG, and Pd-3XDG SACs progressively weaken the metal–support interaction.^30^ The authors observed that this reduction in interaction strength could be attributed to a corresponding increase in the population of the metal–nitrogen anti-bonding states, which, in turn, lead to a destabilization for the metal–support interaction.

Kim *et al.* further observed a progressive weakening of metal–support bonding interaction for period 4 (TM = Sc–Zn), period 5 (TM = Y–Cd), and period 6 (TM = La–Hg) transition metals upon increasing the N-doping of SV sites of the support.^23^ For the different support compositions, the undoped sites, *i.e.*, X = C, exhibited the strongest metal–support interaction, followed by the singly and doubly N-doped sites, with the threefold N-doped site being the least favorable. Interestingly, the effect of N-doping on DV supports differs from that on SV systems. As noted above, Kim *et al.* reported that any N-doping at SV sites weakens the metal–support bonding, however, for DV sites N-doping is generally more favorable.^23^ The authors reported the following order of decreasing stability of N-doping motifs across SV and DV sites: *trans*-2N2C > 3N1C ∼ *cis*(5-ring)-2N2C ∼ 1N3C > *cis*(6-ring)-2N2C > 4C ∼ 4N ∼ 3C > 2N1C, 1N2C > 3N. To probe the electronic structure, the density of states for the most stable configuration, TM-*trans*-2N2CDG, and for the least stable configuration, TM-3NDG was computed, with TM = V, Ni, and Zn. TM-*trans*-2N2CDG SACs adopt square planar like geometries, in which the metal forms four bonds to the N/C-surrounding atoms. In contrast, SV SACs, such as Ni-3NDG, adopt trigonal pyramidal coordination environment, in which the transition metal binds to three N-atoms. In short, planar DV embeddings stabilize the metal center, while SV sites, which force the metal out of the plane of the support, do not.

Gao *et al.* also found that N-doping at SV sites weakened the metal–support interaction, whereas at DV sites N-doping increases this interaction.^21^ They investigated varying degrees of N-doping in TM-3XDG and TM-4XDG supports anchoring period 4 transition metals (TM = Ti–Cu) and noble metals Pt, Ag, and Au. For TM-3XDG, all metals remained positioned above the graphene support, and N-doping showed no significant influence on the elevation height of the metal. In contrast, for DV systems, increasing N-doping reduced elevation height, suggesting that N-doping promotes metal embedding at DV sites. The authors hypothesized that the counteracting effects of N-doping on the stability of TM-3XDG and TM-4XDG may arise from differences in metal–support bonding mechanism and emphasized the need for a comprehensive analysis to further clarify the underlying mechanism.

Mavrikakis *et al.* also reported that the effect of N-doping in the catalyst's stability differs significantly between SV and DV sites.^31^ They systematically examined how varying degrees of N-doping affect the binding interaction of SV and DV sites with various transition metals (TM = Ni, Pd, Pt, Cu, Ag, and Au). Their findings indicated that N-doping generally weakens the binding of transition metal atoms to SV sites, whereas for DV sites it had a stabilizing effect. This counteracting effect originated from the fact that metal atoms cannot be fully positioned within the plane of the SV sites, while DV sites provided sufficient space for incorporation into the plane of the support, leading to a qualitatively different behavior. In particular, the planar geometry of TM-4XDG configurations enabled strong interactions between the metal's d atomic orbitals and the sp^2^-derived orbitals on the N atoms of the support, resulting in strong bonding. Among the configurations studied, partially N-doped vacancies, such as TM-3N1CDG, formed the strongest bonds with transition metals.

Similarly, Yan *et al.*,^32^ when investigating the effect of N-doping on Fe-based SV and DV SACs, observed that the binding energy of the metal to the graphene support became less favorable upon N-doping in SV sites, whereas it became more favorable for DV sites. They concluded that the large variation in bond strengths across different supports, *i.e.*, variation in vacancy size and different degrees of heteroatom doping, suggests that the nature of the support plays a critical role in determining the stability of graphene-based SACs as we have also demonstrated in this review article.

Likewise, Zheng *et al.* found that the introduction of dopant atoms to SV sites is unfavorable, whereas doping can enhance catalyst stability for DV sites.^25^ For period 4 transition metals (TM = Sc–Zn) as well as Pd and Pt, they investigated the effect of B- and N-doping. Introducing B or N in the SV site weakens the metal–support bonding interaction. However, for DV sites, N-doping leads to an increased metal–support binding strength. Upon substitution of three C in the TM-3CDG by three N-dopants, *i.e.*, TM-3NDG, the N atoms in the vacancy site of the support possessed lone-pair electrons. As a result of the additional electrons in the vacancy site for N compared to C, the TM–N antibonding combinations dropped below the Fermi level, which, in turn, weaken the metal–support interaction. When C was replaced by B-dopants, the B atoms each retain one dangling electron pointing towards the center of the vacancy. The resulting bonding situation was similar to N-doping: in both cases, the number of electrons participating in bonding states was reduced relative to the pristine, undoped vacancy, which may explain the weakening of the metal–support interaction upon heteroatom doping. Similar results were found for the DV sites: when the C atoms are replaced by either N or B atoms, less electrons participate in the bonding states resulting in a weaker metal–support interaction. Nevertheless, the stability of the DV SACs increases upon N-doping. The authors ascribed this observation to the fact that the N-doped DV site undergoes less structural deformation upon metal coordination than undoped DV site. This line of reasoning, however, cannot account for the intrinsic metal–support bonding interaction differences reported in other studies, because it is based on the deformations of the pristine supports prior to bonding to the metal center.

Di Valentin *et al.* investigated the impact of N-doping on the structural, electronic, and energetic properties of TM-4XDG SACs, with TM = Fe, Co, Ni, and Cu.^17,33^ In their work, they qualitatively described the bonding mechanism and demonstrated that the pristine TM-4CDG adopted a square planar geometry. Based on this observation, they proposed a metal–support binding mechanism based on crystal and ligand field splitting (Fig. 6). The square planar geometry induces a crystal field splitting in which the d_*xz*_ and d_*yz*_ atomic orbitals of the metal are equally stabilized, followed by the non-bonding d_*z*^2^_ and d_*x*^2^–*y*^2^_ atomic orbitals, while the d_*xy*_ orbital is most strongly destabilized. The d_*xz*_ and d_*yz*_ atomic orbitals of the metal engage in π-type interactions with the p_*z*_ atomic orbitals of the X atoms of the support, whereas the d_*xy*_ atomic orbital forms a σ-type interaction with the sp^2^-hybridized orbitals of the X atoms of the support. A similar splitting scheme is observed for both undoped (X = C) and N-doped (X = N) support, with the latter resulting in structures that are even closer to the ideal square planar complexes compared to the undoped systems. However, the p_*z*_ and sp^2^-hybridized orbitals of N are lower in energy than the analogous orbitals of C, due to the higher effective nuclear charge of the former. Consequently, both the TM–X σ bonding and σ* antibonding orbitals, resulting from the interaction between the metal and the support, are lower in energy for the N-doped support compared to the undoped support. Although the σ* antibonding orbital consistently remains above the Fermi level, its energy is substantially reduced in N-doped SACs. Additionally, all metal d atomic orbitals are, in the case of N-doped SACs, confined within a narrower energy band, indicating a reduced mixing between the metal and the π-system of the graphene support. This suggests that N atoms effectively isolate the transition metal center from the π-system of the graphene support. Based on the analysis of the geometries of the SACs, the authors concluded that N-doping improved local structural symmetry and, hence, the stability of the metal–support interaction.

Similarly, Bao *et al.* reported complementary findings for Fe-4XDG systems, reinforcing the significance of N-doping.^34^ They found that N-doping significantly increases the Fe–support bond strength relative to undoped DV graphene. This computational trend aligned with their experimental findings further supporting the conclusion that N-dopants act as anchoring sites that enhance the stability of isolated Fe atoms in graphene. Likewise, Keifer *et al.* also demonstrated that N-doping improves the stability of Fe-4XDG SACs. This further amplifies the role of N-doping as an effective anchoring strategy.^35^

Moving beyond N-doping, Zhang *et al.* investigated whether other dopants, such as O or mixed N/O configurations, influence the stability of Fe-4XDG systems.^36^ For configurations with two different dopant atoms, such as Fe-2N2ODG, Fe-2N2CDG, and Fe-2O2CDG, the *trans* configuration was found to be more stable than the *cis* configuration. For Fe-*a*N*b*ODG SACs (*a*, *b* = 1, 2, 3; *a* + *b* = 4), increasing N-doping strengthened the Fe–support bonding interaction, leading to an enhanced catalyst stability. In contrast, For Fe-*a*N*b*CDG SACs (*a*, *b* = 1, 2, 3; *a* + *b* = 4), N-doping only slightly altered the binding strength, suggesting that Fe–C and Fe–N bonds exhibit comparable strengths. For Fe-*a*O*b*CDG SACs (*a*, *b* = 1, 2, 3; *a* + *b* = 4), higher degree of O-doping led to lower binding energies, implying that O-doping reduces the catalyst's stability. Overall, these findings demonstrated that N- and C-dopants promote strong Fe anchoring to the DV sites of graphene, whereas O-doping diminishes the stability of Fe–support interaction in Fe-4XDG single-atom catalysts.

Extending to B-doping, Pacchioni *et al.* investigated the stability of TM-1B3NDG, TM-*cis*(6-ring)-2B2NDG, and TM-3B1NDG systems containing period 4 transition metals (TM = Sc–Zn).^37^ By analyzing the formation and binding energies, they found that most catalysts are stable, except for Cr-3B1NDG, Cu-3B1NDG, and all Zn-based systems. In general, the stability of the catalyst decreased with increasing boron doping concentration.

Pašti *et al.* examined the static and dynamic stability of TM-*a*O*b*NDG and TM*-a*S*b*NDG SACs (*a*, *b* = 1, 2, 3; *a* + *b* = 4) incorporating Mn, Fe, and Co.^38^ Across all three transition metals, they observed generally similar characteristics for a given *a*X*b*NDG support. While all TM-*a*O*b*NDG SACs remained square planar, TM-*a*S*b*NDG SACs exhibited a pronounced structural distortion of the graphene support, where the S dopants are protruding out of the plane of the support. By combining bonding energy with Pourbaix diagram analyses, they demonstrated that substituting the nitrogen atoms in TM-4NDG by O or S leads to destabilization of the SACs. Moreover, TM-*a*X*b*NDG SACs with low nitrogen doping exhibited bonding energies less favorable than the corresponding metal cohesion energies, indicating an increased susceptibility to demetallation. Notably, they identified Fe-based SACs for which O- or S-doping can enhance the catalyst's hydrogen evolution reaction activity; however, the majority of these catalysts were predicted to be unstable under electrochemical operating conditions. Consequently, this shows that assessments of catalytic activity is only meaningful when the corresponding catalyst stability, by means of the metal–support interaction, has also been established.

**Fig. 9:**
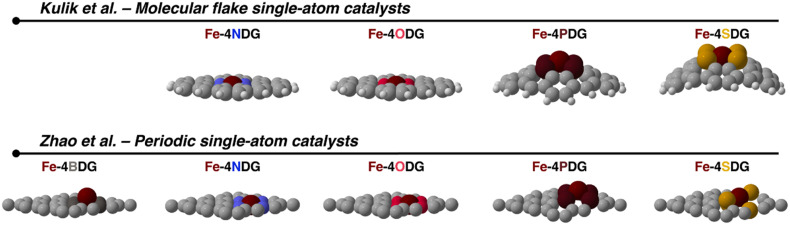
Schematic representation of transition-metal atoms anchored at heteroatom-doped double vacancy graphene sites (Fe-4XDG, where X = B, N, O, P, S). The top row shows the molecular model systems based on the Cartesian coordinates reported by Kulik *et al.*,^39^ whereas the bottom row shows the extended structures based on the Cartesian coordinates from Zhao *et al.*^40^ For period 2 dopants (B, N, and O), distortion only at the transition metal atoms can be observed. In contrast, period 3 dopants (P and S) induce larger structural distortions that affect both the metal center and the dopant atoms.

Further expanding the range of heteroatom dopants, Kulik *et al.* extended this to how different heteroatoms, *i.e.*, N-, O-, P-, and S-doping, influence metal–support bonding in Fe–4XDG (Fig. 9).^39^ Using molecular flakes as model systems for the SACs, they observed that Fe–support bonding was the strongest for N-doping, followed by P-, S-, and lastly O-doping. This trend did not correlate with the TM–X bond lengths: the longest bonds occurred for S, followed by P, whereas N and O exhibited significantly shorter bonds. Doping with period 2 elements (N and O) resulted in planar SACs, while doping with period 3 elements (P and S) induced substantial distortion of the support (Fig. 9). This distortion was attributed to variations in the relative covalent radii of the dopants, whereby the energetic penalty of forming exceptionally short metal–support bonds in the period 3-doped Fe–4XDG SACs was so high that distortion of the graphene support became the preferred. This interpretation was established by constraining the geometry of the period 3-doped SACs to a planar structure; under this constraint, Fe–support bond lengths shortened and bonding weakened. Additionally, compared to analogous molecular catalysts with freely movable ligands, SAC models exhibited much shorter TM–X bond lengths, highlighting the influence of SAC rigidity on the geometry of the catalytically active site.

Beyond Fe-based systems, Zhao *et al.* performed a structural–stability analysis, evaluating geometrical features across a wide range TM-4XDG SACs (TM = Sc–Zn, X = B, N, O, P, and S) (Fig. 9).^40^ They found that for period 2 dopants (N, B, and O) only certain metals were elevated above the plane of the support. For N-doping, only the early transition metals Sc, Ti, and V were lifted out of the graphene plane, with vertical displacements of 1.0–1.3 Å. In contrast, B-doping induced displacements of 1.0–1.6 Å for all transition metals, underscoring the significant role of the dopant in shaping the local metal coordination environment. O-doping produced the largest variability: early and late transition metals exhibited moderate out-of-plane displacement, whereas mid-row transition metals (TM = Fe, Co, Ni) remained planar. In contrast, for period 3 dopants (P and S), distortion was universally observed regardless of the metal center. For the period 3 dopants, structural distortion by means of atom displacement out of the plane of the support occurred for both the metal center and the dopant atom, with P exhibiting a larger degree of elevation than S. The authors concluded that the structural integrity of graphene-based SACs depends strongly on the dopant's atomic radius, with radii similar to carbon being favored. Accordingly, period 2 dopants have atomic radii more similar to carbon than period 3 dopants and, therefore, are more likely to adopt the preferred planar geometry. Moreover, bonds between period 3 elements and carbon are generally longer than the C–C bonds in graphene. To accommodate these longer bonds within the rigid graphene framework, while also coordinating the metal, the period 3 dopants protrude out of the plane of the support. Consequently, period 2 dopants are structurally more suited to form stable SACs than period 3 dopants.

Tang *et al.* conducted a study on TM-4XDG SACs incorporating transition metals from period 4 (TM = Sc–Zn), period 5 (TM = Y–Cd), and period 6 (TM = Hf–Au), combined with dopants X = B, N, O, P, and S.^41^ They found that oxygen doping generally reduced the thermodynamic stability of the catalyst, and increasing the degree of oxygen doping further destabilized the SACs. In contrast to the priory discussed studies, these authors concluded that the undoped TM-4CDG exhibited superior structural stability, which they hypothesize to originate from the electronic structure modifications within the coordination environment.

In summary, heteroatom doping has a fundamentally different effect on the stability of SV and DV SACs, that is, for TM-3XDG compared to TM-4XDG, respectively. For SV sites, heteroatom doping generally weakens metal–support interactions, whereas for DV sites such doping can, depending on the heteroatom, significantly enhance the binding between metal and support. This contrast arises from their distinct coordination environments: unlike SV SACs, DV SACs can adopt planar geometries that favor stronger bonding, as discussed in Section 2. However, the quantitative origin of the strengthened bonding interactions in DV SACs remains a matter that needs to be studied in more detail. Among period 2 dopants, most studies conclude that N-doping is particularly effective in stabilizing DV SACs, with the partially substituted configuration *trans*-TM-2N2CDG emerging as the most favorable. In contrast to N-doping, O-doping consistently reduces the stability of DV SACs and increasing the degree of O-doping further destabilizes the complex. Summarizing, period 2 dopants (N, B, and O) tend to preserve planarity and, thereby, enable stronger metal–support interactions, while period 3 dopants (P and S) introduce geometric distortion and, hence, weaken the metal–support binding due to the heteroatom's large covalent radii and, consequently, longer TM–X bonds. In all, N-doped graphene supports can be identified as preferred anchoring sites for strong metal–support interactions, highlighting the preservation of planarity as a key criterion when selecting dopant atoms for DV sites.

## 4. Changing the catalytically active transition metal

**Fig. 10:**
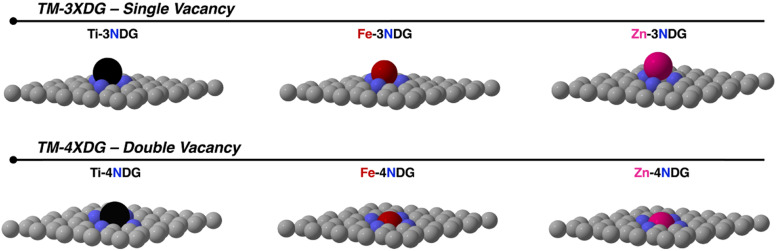
Schematic representation of transition-metal atoms anchored at single and double vacancies graphene sites (TM-3NDG and TM-4NDG). For TM-3NDG sites, the transition metal always resides above the graphene support, whereas in TM-4NDG systems, the transition metal can reside above or in the same plane, depending on the nature and size of the transition metal.

Thus far, we have examined how changes in the graphene support, that is, vacancy size and heteroatom doping, influence the stability of SACs. Now, we turn to the effect of varying the catalytically active transition metal atom on the metal–support binding interaction. For catalysts with an SV graphene support, Sánchez-Portal *et al.* investigated TM-3CDG SACs containing period 4 transition metals (TM = Sc–Zn) as well as noble metals Ag and Au.^42^ All metals adopted typical trigonal pyramidal geometries, with the metal positioned above the graphene plane as earlier discussed (Fig. 10). By examining the bonding mechanism, the authors found that Ti exhibited the strongest bond to the support, which they attributed to the full occupation of all bonding states. Following this rationale, one would expect a decrease in binding strength along period 4, as the nonbonding and, subsequently, antibonding states became consistently more occupied. However, this trend did not hold: the weakest metal–support bond occurred for Cr-3CDG and Mn-3CDG, whereafter the bond strengthens again going to Co-3CDG. The observed behavior was explained by two competing effects. First, the 3d atomic orbitals of the metal lowered in energy as the atomic number increases. This stabilization made the 3d atomic orbitals becoming progressively more filled, resulting in a reduced number of TM_3d_–C_sp^2^_ states near the Fermi level and hence a reduction in binding strength. Second, moving from Mn to Zn, the metal–support bond length decreased by approximately 0.1 Å, strengthening the metal–support interaction and correspondingly increasing the catalysts stability.

Meng *et al.* investigated the bonding interaction between late period 4 transition metals (TM = Fe–Zn) and the support in TM-3CDG SACs.^43^ They also reported the typical trigonal pyramidal SV geometry, with the transition metal positioned above the graphene plane, consistent with the larger atomic radii of the metals relative to the C atoms in the support (Fig. 10). In contrast to the work of Sánchez-Portal *et al.*, a slightly different trend in binding energies was observed: from Fe-3CDG to Ni-3CDG, the metal–support binding strength decreased, due to the increasing occupation of the metal's 3d atomic orbitals, which weakened the interaction with the sp^2^-hybridized orbitals of C. Density of states calculations of the 3CDG showed prominent peaks near the Fermi level that originate from sp^2^-hybridized orbitals of C. Upon bonding with the metal, a downshift and broadening of both the metal's 3d atomic orbitals and the sp^2^-hybridized orbitals of C was observed, evidencing interactions between the two orbitals. For Cu-3CDG and Zn-3CDG, their occupied 3d atomic orbitals exhibited poor compatibility with sp^2^-hybridized orbitals of C in the SV, yielding less stabilizing metal–support interactions and, correspondingly, larger out-of-plane displacements and longer bond lengths.

Extending these observations beyond period 4 metals, Dai *et al.* examined late transition and post-transition metals, namely Pt, Pd, Au and Sn, in TM-3CDG SACs.^44^ They concluded that all four metals were able to bind to SV sites, forming strong bonds with undercoordinated carbon atoms. Consistent with their larger atomic radii relative to carbon, in their optimal geometries, the metal atoms were positioned above the graphene plane. Sn binds slightly more strongly than Au; however, both exhibited significantly weaker binding compared to Pd and Pt. Metals with an undersaturated d atomic orbitals, like Pt, form stronger covalent bonds to the SV than other metals, implicating that the undersaturated d atomic orbitals must play a crucial role in the strength of the interaction between the metal and the support.

Piotrowski *et al.* investigated the bonding interaction in TM-3CDG between the metals Co, Ni, Rh, Pd, Ir, and Pt and the support.^45^ All studied metals anchored directly to the SV site, with metal–carbon distances that were indicative of covalent TM–C bonding. The corresponding binding energies were highly stabilizing, demonstrating that the SV site efficiently stabilized these isolated single atoms. Electronic structure analysis showed that the sp^2^-hybridized orbitals of C in the SV strongly mix with metal's d atomic orbitals, promoting charge transfer from the metal to the C atoms of the support. Across the examined metals, increasing the atomic number correlated well with the moderate increase in TM–C bond distance and hence weakening of the metal–support bond strength. This is consistent with the larger atomic radii, which reduces the ability to be anchored within the SV sites.

Nieminen *et al.* analyzed the metal–support interaction in both TM-3CDG and TM-4CDG, with period 4 transition metals (TM = Sc–Zn) and noble metals Pt and Au.^46^ All transition metals formed covalent bonds in TM-3CDG, adopting a trigonal pyramidal geometry where the metal atom is positioned above the support (Fig. 10). The metal–support interaction was generally strong, except for metals with nearly filled d atomic orbitals, that is, Cu and Zn, which exhibited weaker interactions due to increased population of the antibonding combinations. The TM–C bond length decreased from Sc to Fe, in line with the reduced atomic size, then increased again as the metal–support bonding interaction weakens. The TM-4CDG SACs showed a similar trend in bond strength across the series. Band structure analysis revealed strongly mixing between the metal's d atomic orbitals and the sp^2^-hybridized orbitals of C. To rationalize these observations, the authors analyzed the bonding mechanism. As case in point, they considered Ti-3CDG, where Ti had four valence electrons, which allow for the formation of three σ bonding combinations and one π bonding combination with the support, resulting into three σ bonds and one π bond. Ti's tetravalency made it an ideal substitute for C, giving the most stabilizing bonding energy among all investigated metals. However, even though Ti was often considered the best replacement for the missing C atom, its larger atomic radius prevented the formation of a planar geometry. A similar reasoning applied to Ti-4CDG sites, where four σ bonds and one potential π bond exist, though the metal–carbon π interaction was weaker due to greater spatial separation between the interaction Ti and the support.

Turning to TM-4XDG SACs, Di Valentin *et al.* examined both undoped (X = C) and N-doped (X = N) systems containing Fe, Co, Ni, and Cu.^17,33,47^ They provided a qualitative description of the bonding mechanism (Fig. 6), as discussed in the previous section. Their analysis revealed that Cu-4XDG exhibited the weakest interaction with the support, because its 3d atomic orbitals lie further below the Fermi level compared to the other transition metals investigated, thereby weakening the bonding interaction with the sp^2^-hybridized orbitals of the support.

**Fig. 11:**
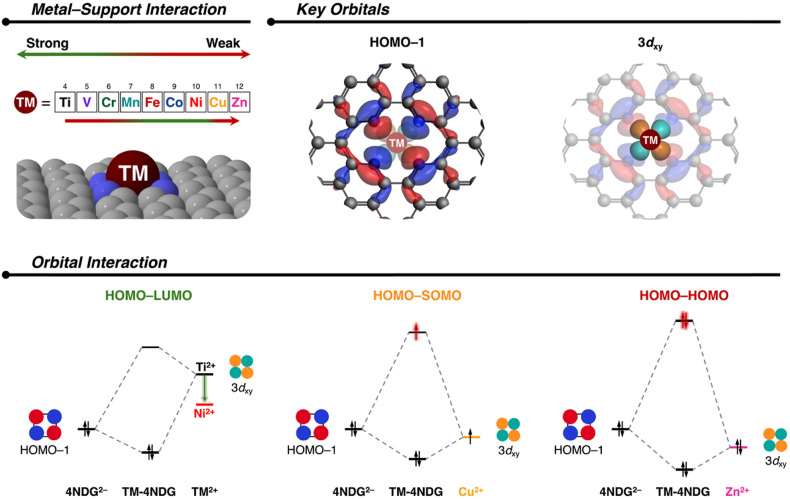
Schematic overview illustrating how the stability of TM-4NDG systems (TM = Ti, V, Cr, Mn, Fe, Co, Ni, Cu, and Zn) varies with the nature of the transition metal. The top-left panel summarizes the trend in metal–support bond strengths across the period 4 series. The top-right panel highlights the key orbitals governing this interaction: the HOMO–1 of the 4NDG support and the metal 3d_*xy*_ atomic orbital. The bottom panel presents schematic molecular orbital diagrams. From left to right, Ti to Ni exhibit an increasingly stabilizing HOMO–LUMO interaction; Cu showed a partially repulsive HOMO–SOMO interaction; and Zn displayed a fully repulsive HOMO–HOMO interaction. Adapted from van Dam and Vermeeren,^48^ published under the Creative Commons Attribution 4.0 License (CC BY 4.0).

Van Dam and Vermeeren extended the qualitative description of the bonding interactions in TM-4NDG SACs to a quantitative framework,^48^ using the activation strain model,^49^ Kohn–Sham molecular orbital theory,^50^ and canonical energy decomposition analysis (Fig. 11).^51^ The authors demonstrated that the strength of the interaction between the metals and the support along period 4 (TM = Ti–Zn) was governed by two intrinsic characteristics of the transition metal: (i) the increasing effective nuclear charge; and (ii) the progressive filling of the 3d atomic orbitals. The dominant bonding mechanism involved the interaction between the TM's 3d_*xy*_ orbital and the nitrogen lone pairs on 4NDG. From Ti-4NDG to Ni-4NDG, the rising effective nuclear charge of the transition metal effectively lowered the energy of the 3d_*xy*_ orbital, reducing the HOMO–LUMO gap and, thereby, strengthening this interaction. Notably, the natural energy decomposition analysis (NEDA) performed by Nova *et al.* revealed a similar trend, showing progressively strengthening of the charge transfer interactions between the metal and support from Mn-4NDG to Fe-4NDG to Co-4NDG.^52^ For Cu-4NDG and Zn-4NDG, the bond weakened as the 3d_*xy*_ orbital becomes increasingly occupied, singly for Cu and doubly for Zn, shifting the interaction from favorable HOMO–LUMO to less favorable HOMO–SOMO and, ultimately, unfavorable HOMO–HOMO interaction. The ideal stable TM-4NDG SAC was found for transition metals positioned sufficiently far to the right in period 4 for the increased nuclear charge to stabilize the 3d_*xy*_ atomic orbital, yet not so far that this atomic orbital becomes occupied.

Interestingly, Meng and Zhong also reported that Co-4NDG and Ni-4NDG exhibited the highest stability in their dynamic stability analysis.^53^ Specifically, they examined TM-4NDG SACs comprising transition metals from period 4 (TM = Cr–Ni) under electrochemical operating conditions across a range of pH values and applied potential, while also accounting for structural modifications associated with substrate vacancies. Based on Pourbaix diagram analyses for each catalyst, both in the absence and presence of adsorbates, Co-4NDG and Ni-4NDG were identified as the overall most stable catalysts. Notably, the stability of Co-4NDG is further enhanced in the presence of adsorbates (H, OH, or OOH). Fe-4NDG is predominantly stable when O is adsorbed but can also remain stable with H or OOH adsorption under specific pH conditions and potentials. In contrast, Mn-4NDG exhibits only limited stability, whereas Cr-4NDG was determined to be unstable under all investigated conditions. This again demonstrates that having strong metal–support interactions, such as in Co-4NDG and Ni-4NDG, will lead to SACs that are stable under electrochemical operating conditions.

Pašti *et al.* reported similar findings for the stability of TM-4NDG SACs under electrochemical conditions.^54^ They considered transition metals from period 4 (TM = Mn–Cu), period 5 (TM = Ru–Ag), and selected metals from period 6 (TM = Ir–Au). Through thermodynamic analysis, they demonstrated that catalyst stability generally increases across a given period but decreases markedly for the coinage metals. This trend was attributed to the relatively weak interaction between coinage metals and the 4NDG support. Regarding pH effects, certain metal centers, such as Mn, Co, and Ni, were predicted to dissolve under acidic conditions, whereas others (TM = Ru, Rh, Ir, Pd, Pt) are expected to remain stable across the entire pH range.

Soldano *et al.* reported a different trend for SAC stability upon varying the transition metal.^22^ They found for both TM-3XDG and TM-4XDG SACs, that early transition metals (TM = Sc–Ti) bind most strongly, mid-series metals (TM = Cr–Co) show intermediate binding, and late metals (TM = Ni–Zn) bind most weakly, independent of the support. For TM-3CDG SACs, Ti has 3d atomic orbitals near the Fermi level, enabling efficient mixing with both the p_*z*_ and sp^2^-hybridized orbitals of C. In contrast, Zn has lower-lying 3d atomic orbitals with limited overlap with the sp^2^-hybridized orbitals of C, explaining its weaker interaction. For TM-4CDG, in contrast, the trend in binding interaction is dominated by structural effects: Sc-4CDG adopts a more elevated geometry, while Cu-4CDG binds nearly in-plane of the support, promoting better overlap with the C atoms of the support. Consequently, for Sc-4CDG, the d_*xz*_ and d_*yz*_ atomic orbitals that are perpendicular to the plane of the support dominate the bonding mechanism, whereas for Cu-4CDG the in-plane d_*xy*_ atomic orbital binds more effectively with the support stabilizing the complex. Going from Cr-4CDG to Co-4CDG, an intermediate binding strength in observed, because stabilizing electronic and destabilizing structural contributions largely compensate each other. Ni-4CDG, Cu-4CDG, and Zn-4CDG are all nearly planar, but bonding weakens across the series, revealing that the geometry does not always govern the trend metal–support interaction. Along this series, the metal–support antibonding state became increasingly occupied, weakening the interaction, as demonstrated using quantitatively analyses by van Dam and Vermeeren.^48^ In addition, for Zn-4CDG, there is an unfavorable alignment between the 3d atomic orbitals of Zn and the sp^2^-hybridized orbitals of C further weakening this bond.

Extending the analysis of metal variation in TM-4NDG SACs, Wang *et al.* investigated Ba, Mg, and Ti anchored to four nitrogen-doped graphene and reported that the binding strength decreases from Ti-4NDG to Ba-4NDG to Mg-4NDG.^55^ Although all three metals were located at the center of the 4NDG DV site, forming four TM–N bonds, Mg was the only metal positioned in the graphene plane, whereas Ti and Ba resided above the plane with elevation heights of 0.89 Å and 1.72 Å, respectively. Density of states analysis showed that for all three metals, the C 2p and N 2p atomic orbitals of the support contribute significantly. However, the electronic structure of Ti-4NDG differs in nature from Mg-4NDG and Ba-4NDG. For Ba and Mg, N 2p peaks at the Fermi level indicate TM–N mixing and bond formation, suggesting strong interaction between these metals and the N-doped graphene support. The symmetric spin-up and spin-down DOS of the Ba 5p and Mg 2p atomic orbitals result in no net magnetic moments. In contrast, the Ti 3d atomic orbitals are asymmetric, especially near the Fermi level, indicating large magnetic moments. Spin-density analysis further revealed strong localization on Ti metal center and the coordinating N atoms, suggesting that Ti stabilizes N sites and N atoms play a beneficial role in magnetic moment localization through by strong Ti–N interaction. Additionally, Bader charge analysis showed that Ti and the C atoms adjacent to N dopants carry positive charge due to the higher electronegativity of N, which contributes to the greater stability of Ti-4NDG relative to Ba-4NDG and Mg-4NDG.

Extending to beyond N-doping, Tang *et al.* systematically examined TM-4XDG SACs to assess the effect of the transition metal in conjunction with heteroatom doping on the SAC's stability.^41^ Their dataset spanned period 4 (TM = Sc–Zn), period 5 (TM = Y–Cd), and period 6 (TM = Hf–Au) transition metals, combined with B, N, O, P, and S dopants. They reported that the period 3 metals Sc, Ti, V, Cr, and Mn, as well as selected period 4 and period 5 metals Y, Zr, Nb, and Hf, exhibited reasonably stable metal–support interactions. In addition, Fe, Co, Ni, Cu, Re, Pd, Ir, and Pt not only showed strong metal–support interactions, but also promoted electrochemical stability. A further analysis of the origin of the trends in metal–support interaction was, however, not performed. Yang *et al.*, on the other hand, investigated N,S-co-doped TM-4XDG SACs containing period 4 (TM = Sc–Zn), period 5 (TM = Y–Cd), and period 6 (TM = Lu–Hg) elements.^56^ Across all three periods, bond energies became less stabilizing with increasing atomic number. Moreover, dissolution potentials become larger with increasing atomic number, indicating that demetallation is less thermodynamically favorable. Accordingly, SACs containing early transition metals were assessed to be less stable.

In conclusion, changing the catalytically active transition metal atom has a drastic effect on the strength of the metal–support interaction and hence the stability of SACs under operating conditions. For all period 4 metals (TM = Sc–Zn) in TM-3CDG supports, metal–support bonding is established, yet the trends in bond strengths do not consistently agree across different studies. Identifying the sources of these discrepancies is challenging, largely because essential structural details, such as Cartesian coordinates, spin states, and oxidation states, are frequently not reported. Nevertheless, it has generally been agreed to that late transition metals with largely or fully occupied d atomic orbitals, such as Cu or Zn, bind the weakest to the support. Furthermore, Ti is commonly expected to bind well due to its tetravalency, which enables it to effectively substitute the missing C atom in the SV site of the support. However, its larger atomic radius compared to carbon still prevents the formation of a planar coordination geometry. For the popular TM-4NDG SACs, Fe, Co, and Ni are generally expected to bind strongly to the support. Quantitative analyses indicate that this preference emerges for two reasons. First, these transition metals sit far enough to the right in the periodic table that their effective nuclear charge is large enough to stabilize the 3d_*xy*_ atomic orbital. Second, the 3d_*xy*_ atomic orbital remains unoccupied, enabling a favorable HOMO–LUMO-type interaction between the metal and the support. This effect is not only limited to N-doped graphene, as strong metal–support interactions for Fe, Co, and Ni are also found when using other dopants, such as, B, O, P, and S.

## 5. Conclusion and outlook

In this review, we discuss three strategies to tune the metal–support interactions in heteroatom-doped graphene-based single-atom catalysts, based on insights obtained from quantum chemical calculations: the influence of vacancy size in graphene, the role of heteroatom doping, and the effect of varying the transition metal center. Regarding vacancy size, the intrinsic geometric limitations of single vacancy (SV) sites prevent in-plane anchoring of transition metal atoms, as the radii of metal atoms generally exceed that of carbon. In contrast, the larger coordination cavity of double vacancy (DV) sites consistently supports (near) in-plane embedding of the metal atom and, therefore, facilitates stronger metal–support bonding. The weaker metal–support interactions of TM-3XDG SACs compared with TM-4XDG SACs, therefore, stem from the fundamental differences in their local coordination environments. The planarity of the active center in TM-4XDG SACs is widely regarded as a key factor contributing to their enhanced stability, compared to the trigonal pyramidal TM-3XDG structures.

The difference between the SV and DV anchoring sites becomes even more apparent when heteroatom doping is involved. Introducing a dopant atom at an SV site decreases the metal–support interactions of the resulting SAC, whereas appropriate dopants at DV sites can enhance metal–support interactions. Among commonly studied heteroatoms, only N-doping is found to have a stabilizing effect on TM-4XDG SACs, with partial doping motifs, such as *trans*-2N2CDG, being preferred. For other dopants, period 2 elements, such as B and O, tend to weaken the metal–support interactions in TM-4XDG SACs less severely than period 3 dopants. Period 2 dopants preserve the planarity of the coordination environment, whereas period 3 dopants introduce geometric distortions due to their larger atomic radii and preference for longer TM–X bonds. Taken together, N dopant incorporation at DV sites appears to be the most promising strategy to improve metal–support interactions and, thereby, the catalyst's stability.

Changing the catalytically active transition metal atom has a drastic effect on the strength of the metal–support interaction. For the TM-3CDG, it was generally found that the early transition metal Ti binds the strongest to the support. For TM-4NDG systems, on the other hand, Fe, Co, and Ni consistently exhibit the strongest binding among the period 4 metals, owing to their favorable HOMO–LUMO type interactions with the graphene support. This latter behavior can be attributed to a balance between sufficiently high effective nuclear charge, which stabilizes the 3d_*xy*_ atomic orbital, and an electron count that prevents this atomic orbital from becoming fully occupied. Similarly, strong metal–support interactions are expected for Fe, Co, and Ni when coordinated to dopant atoms such as B, O, P, and S.

Taken altogether, the metal–support interactions in transition metal-anchored heteroatom-doped graphene single-atom catalysts emerge from a subtle interplay between vacancy size and the nature of both the dopant and transition metal. Current evidence points to DV-based, N-doped TM-4XDG motifs with Fe, Co, or Ni as particularly promising, as they adhere to two key principles for maintaining or enhancing metal–support interactions: (i) preservation of planarity, facilitated by larger DV cavities and smaller heteroatom dopants, and (ii) avoidance of late d^9^ and d^10^ transition metals, which would otherwise lead to occupation of antibonding orbitals weakening the metal–support interaction.

Nevertheless, the presence of scattered and sometimes contradictory trends in the literature highlight that our present understanding of the underlying stabilizing interactions remains incomplete. Furthermore, we show that the metal–support bond strength can depend strongly on how the energetics are evaluated. Opposing trends in literature can arise because some frameworks incorporate the energetics of deforming the vacancy site prior to metal coordination. While this perspective captures an additional contribution to the overall energetics, it naturally leads to stability trends that differ from analyses focused exclusively on the intrinsic stability of the final metal–support structure. Obtaining a more complete picture of the metal–support interactions in SACs will, therefore, require systematic and consistent computational studies that explicitly analyze and compare vacancy sizes, effects of doping and transition metals, and metal–support bonding mechanisms, and that are supported by transparent reporting of structural and electronic details, including Cartesian coordinates of the computed structures as well as clear descriptions of the employed spin states and oxidation states. Such efforts will not only clarify the physical origin of metal–support interactions in these systems but also tuning strategies for optimizing the intrinsic stability of graphene-based SACs.

Finally, we want to stress that stability alone, by means of metal–support interactions, cannot dictate catalyst design. Achieving an optimal balance between stability and reactivity is essential; thus, a moderate reduction in stability may be acceptable when paired with substantially increased catalytic activity. Therefore, the use of alternative vacancy sizes, dopants, and metals should *a priori* not be dismissed, although their potential to reduce catalyst stability must be carefully considered.

## Conflicts of interest

There are no conflicts to declare.

## Data Availability

No new data are generated or analyzed as part of this review.

## References

[cit1] (d) Yuk S. F. , Collinge G., Nguyen M.-T., Lee M.-L., Glezakou V.-A. and Rousseau R., Single-Atom Catalysis: An Analogy between Heterogeneous and Homogeneous Catalysts. In Advanced Heterogeneous Catalysts Volume 2: Applications at the Single-Atom Scale, 2020, pp, 1–15

[cit2] Zhang W., Fu Q., Luo Q., Sheng L., Yang J. (2021). JACS Au.

[cit3] Kim I. H., Lim J., Kim S. I. (2021). Acc. Mater. Res..

[cit4] Bellunato A., Arjmandi Tash H., Cesa Y., Schneider G. F. (2016). Chem. Phys. Chem..

[cit5] Zhang T., Zhu L., Yuan S., Wang J. (2013). Chem. Phys. Chem..

[cit6] Rong X., Wang H.-J., Lu X.-L., Si R., Lu T.-B. (2020). Angew. Chem., Int. Ed..

[cit7] van Dam A. N., Vermeeren P. (2026). J. Phys. Chem. C.

[cit8] Zhang Z., Huang X., Xu H. (2021). ACS Appl. Mater. Interfaces.

[cit9] Zhao C.-X., Li B.-Q., Liu J.-N., Zhang Q. (2021). Angew. Chem., Int. Ed..

[cit10] Chen Z., Vorobyeva E., Mitchell S., Fako E., Ortuño M. A., López N., Collins S. M., Midgley P. A., Richard S., Vilé G., Pérez-Ramírez J. (2018). Nat. Nanotechnol..

[cit11] Shu S., Song T., Wang C., Dai H., Duan L. (2024). Angew. Chem., Int. Ed..

[cit12] Bae G., Han S., Oh H.-S., Choi C. H. (2023). Angew. Chem., Int. Ed..

[cit13] Di Liberto G., Giordano L., Pacchioni G. (2024). ACS Catal..

[cit14] Bai X., Zhao X., Zhang Y., Ling C., Zhou Y., Wang J., Liu Y. (2022). J. Am. Chem. Soc..

[cit15] Su Y.-Q., Zhang L., Wang Y., Liu J.-X., Muravev V., Alexopoulos K., Filot I. A. W., Vlachos D. G., Hensen E. J. M. (2020). npj Comput. Mater..

[cit16] Liu K., Zhao X., Ren G., Yang T., Ren Y., Lee A. F., Su Y., Pan X., Zhang J., Chen Z., Yang J., Liu X., Zhou W. X. I. T., Luo J., Zeng C., Matsumoto H., Liu W., Jiang Q., Wilson K., Wang A., Qiao B., Li W., Zhang T. (2020). Nat. Commun..

[cit17] Baby A., Trovato L., Di Valentin C. (2021). Carbon.

[cit18] Odkhuu D. (2016). Phys. Rev. B.

[cit19] Jean Y. , Molecular Orbitals of Transition Metal Complexes, (English version), Oxford University Press, 2005, pp. 77–79

[cit20] Malola S., Häkkinen H., Koskinen P. (2009). Appl. Phys. Lett..

[cit21] Yang W., Xu S., Ma K., Wu C., Gates I. D., Ding X., Meng W., Gao Z. (2020). Nano Mater. Sci..

[cit22] Morgan N. A. B. , Cipollone A., López A. S., Mariscal M. M. and Soldano G. J., Available at SSRN: https://ssrn.com/abstract=5953638

[cit23] Ha M., Kim D. Y., Umer M., Gladkikh V., Myung C. W., Kim K. S. (2021). Energy Environ. Sci..

[cit24] Tang Y., Zhou J., Shen Z., Chen W., Li C., Dai X. (2016). RSC Adv..

[cit25] Zhang X., Yu S., Chen H., Zheng W. (2015). RSC Adv..

[cit26] Li J., Jiang Y.-f, Wang Q., Xu C.-Q., Wu D., Banis M. N., Adair K. R., Doyle- Davis K., Meira D. M., Finfrock Y. Z., Li W., Zhang L., Sham T.-K., Li R., Chen N., Gu M., Li J., Sun X. (2021). Nat. Commun..

[cit27] Alherz A. W., Alsunni Y. A. (2026). Energy Fuels.

[cit28] Menga D., Low J. L., Li Y.-S., Arčon I., Koyutürk B., Wagner F., Ruiz-Zepeda F., Gaberšček M., Paulus B., Fellinger T.-P. (2021). J. Am. Chem. Soc..

[cit29] Chen W., Tang Y., Zhang H., Shi J., Wang Z., Cui Y., Teng D., Li Z., Dai X. (2022). Physica E.

[cit30] Ziat S., Brix F., Tsaturyan A., Kierren B., Gaudry E. (2026). J. Phys. Chem. Lett..

[cit31] Kropp T., Rebarchik M., Mavrikakis M. (2019). Catal. Sci. Technol..

[cit32] Gao Z.-Y., Yang W.-J., Ding X.-L., Lv G., Yan W.-P. (2018). Phys. Chem. Chem. Phys..

[cit33] Perilli D., Breglia R., Di Valentin C. (2022). J. Phys. Chem. C.

[cit34] Deng D., Chen X., Yu L., Wu X., Liu Q., Liu Y., Yang H., Tian H., Hu Y., Du P., Si R., Wang J., Cui X., Li H., Xiao J., Xu T., Deng J., Yang F., Duchesne P. N., Zhang P., Zhou J., Sun L., Li J., Pan X., Bao X. (2015). Sci. Adv..

[cit35] Kattel S., Atanassov P., Kiefer B. (2014). Phys. Chem. Chem. Phys..

[cit36] Han M., Huang Y., Zhang H. (2022). Phys. Chem. Chem. Phys..

[cit37] Giri S., Misra D., Di Liberto G., Pacchioni G. (2026). Adv. Theory Simul..

[cit38] Ritopecki M. S., Dobrota A. S., Skorodumova N. V., Pašti I. A. (2022). Nanomaterials.

[cit39] Jia H., Nandy A., Liu M., Kulik H. J. (2022). J. Mater. Chem. A.

[cit40] Gallagher C., Kothakonda M., Zhao Q. (2025). Phys. Chem. Chem. Phys..

[cit41] Huang Z., Yan Q., Tang Q. (2026). Chem. Commun..

[cit42] Santos E. J. G., Andrés A., Sánchez-Portal D. (2010). New J. Phys..

[cit43] Chu M., Liu X., Sui Y., Luo J., Meng C. (2015). Molecules.

[cit44] Tang Y., Yang Z., Dai C. (2011). J. Chem. Phys..

[cit45] Morais W. O., Felix J. P. C., da Silva G. R., de Oliveira Bastos C. M., Dias A. C., Flores E. M., Rêgo C. R. C., da Silva Ramos de Sousa V., Guedes-Sobrinho D., Piotrowski M. J. (2025). Sci. Rep..

[cit46] Krasheninnikov A. V., Lehtinen P. O., Foster A. S., Pyykkö P., Nieminen R. M. (2009). Phys. Rev. Lett..

[cit47] Breglia R., Perilli D., Di Valentin C. (2023). Mater. Today Chem..

[cit48] van Dam A. N., Vermeeren P. (2025). Chem. – Eur. J..

[cit49] Bickelhaupt F. M., Houk K. N. (2017). Angew. Chem., Int. Ed..

[cit50] (b) Albright T. A. , Burdett J. K. and Whangbo M.-H., Orbital Interactions in Chemistry, John Wiley & Sons, Inc., 2013

[cit51] (a) Hamlin T. A. , Vermeeren P., Fonseca Guerra C. and Bickelhaupt F. M., in Complementary Bonding Analysis, ed S. Grabowsky, De Gruyter, Berlin, 2021, pp. 199–212

[cit52] Denjean A. E. F., Balcells D., Nova A. (2026). J. Mater. Chem. A.

[cit53] Meng Y., Zhong L. (2026). Angew. Chem., Int. Ed..

[cit54] Dobrota A. S., Skorodumovam N. V., Mentus S. V., Pašti I. A. (2022). Electrochim. Acta.

[cit55] Liu L.-L., Chen C.-P., Zhao L.-S., Wang Y., Wang X.-C. (2017). Carbon.

[cit56] Liu J.-H., Sun Y., Jiang X., Jiang H., Wang R., Wang Y., Ye H., Chen X., Yang L.-M. (2026). Fuel.

